# Novel roles of ammonia in physiology and cancer

**DOI:** 10.1093/jmcb/mjaf007

**Published:** 2025-03-06

**Authors:** Guantong Chen, Chenxi Wang, Shuo Huang, Shibo Yang, Qiyuan Su, Yige Wang, Weiwei Dai

**Affiliations:** Department of Biosciences and Bioinformatics, School of Science, Xi'an Jiaotong-Liverpool University, Suzhou 215123, China; Suzhou Municipal Key Lab for Metabolic Syndrome Drug Research, Xi'an Jiaotong-Liverpool University, Suzhou 215123, China; Department of Biosciences and Bioinformatics, School of Science, Xi'an Jiaotong-Liverpool University, Suzhou 215123, China; Department of Biosciences and Bioinformatics, School of Science, Xi'an Jiaotong-Liverpool University, Suzhou 215123, China; Department of Biosciences and Bioinformatics, School of Science, Xi'an Jiaotong-Liverpool University, Suzhou 215123, China; Suzhou Municipal Key Lab for Metabolic Syndrome Drug Research, Xi'an Jiaotong-Liverpool University, Suzhou 215123, China; Suzhou Municipal Key Lab for Metabolic Syndrome Drug Research, Xi'an Jiaotong-Liverpool University, Suzhou 215123, China; Department of Biosciences and Bioinformatics, School of Science, Xi'an Jiaotong-Liverpool University, Suzhou 215123, China; Suzhou Municipal Key Lab for Metabolic Syndrome Drug Research, Xi'an Jiaotong-Liverpool University, Suzhou 215123, China

**Keywords:** ammonia, mTOR, urea cycle, glutamine synthetase, tumor microenvironment

## Abstract

Ammonia, traditionally recognized as a toxic nitrogen waste product, has recently emerged as a significant player in diverse physiological processes and implicated in cancer biology. This review article provides an overview of the multifaceted impact of ammonia on cellular signaling pathways, energy metabolism, and tumor microenvironment dynamics, in particular its novel roles in neurotransmission, metabolic homeostasis, cancer cell proliferation, and immune modulation. Notably, ammonia accumulates within the tumor microenvironment, promoting nonessential amino acid synthesis, stimulating mTORC1 activation, promoting lipid synthesis, and impairing various immune cell functions, thereby promoting tumor progression. Furthermore, the potential dual role of ammonia as a tumorigenic factor and a cancer therapeutic target is discussed, shedding light on its complex regulatory mechanisms and clinical implications. This timely review aims to deepen our understanding of the emerging physiological and pathological roles of ammonia, offering valuable insights into its significance as a potential target for diagnostic and therapeutic interventions in cancer and beyond.

## Roles of ammonia in physiological conditions

Ammonia is primarily produced in the gut as a byproduct of protein metabolism and is normally detoxified by the liver. In physiological conditions, mammals convert toxic ammonia into urea via the urea cycle, predominantly in the liver, to prevent neurotoxicity (DeBerardinis and Cheng, 2010, Tapper et al., 2015). Deficiencies in urea cycle enzymes (UCEs), such as carbamoyl phosphate synthetase 1 (CPS1), can result in hyperammonemia, leading to severe neurological deficits. In the brain, where the urea cycle is inactive, glutamine synthetase (GS) helps maintain low ammonia levels, and disruptions in this system can cause cerebral damage and cognitive impairment (Kim et al., 2017; Dai et al., 2022).

### Ammonia homeostasis in physiological conditions

**Figure 1:**
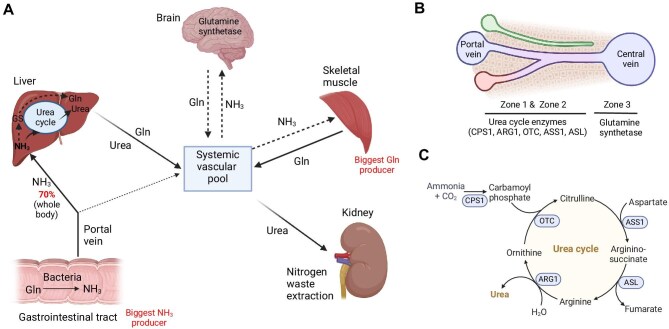
Ammonia metabolism in mammals. (**A**) Whole-body ammonia metabolism in different organs. (**B**) Localization of UCEs and GS in liver zonation. (**C**) Urea cycle diagram and UCEs. ASL, argininosuccinate lyase; ASS1, argininosuccinate synthetase. Created in BioRender.

**Figure 2:**
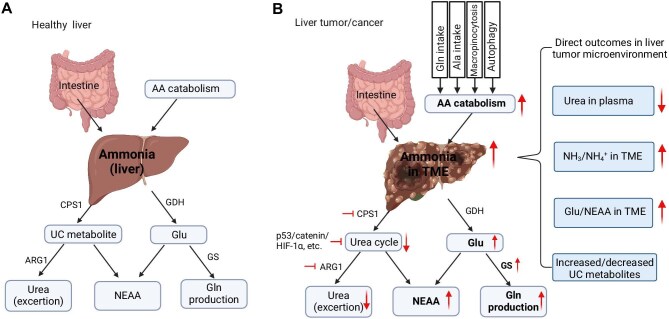
Comparison of ammonia metabolism in healthy liver and the liver TME. (**A**) Ammonia homeostasis in healthy liver. (**B**) Ammonia accumulation in liver tumor/cancer and related outcomes. AA, amino acid; UC, urea cycle. Created in BioRender.

In mammalian cells, ammonia production arises from the catabolism of amino acids during the hydrolysis of dietary and tissue proteins. Nevertheless, the gastrointestinal microbiome in mammals emerges as the primary contributor to ammonia generation (Figure 1A). As elevated ammonia levels in the bloodstream may cause neurotoxicity and seizure, mammals mainly employ the urea cycle in the liver to convert neural toxic ammonia into nontoxic urea, which is subsequently transported to the kidneys for excretion in the urine (Figure 1B and C; DeBerardinis and Cheng, 2010; Häberle, 2011; Tapper et al., 2015). This process serves a vital role for maintaining low levels of ammonia in the bloodstream. Enzyme deficiencies within the urea cycle, exemplified by CPS1, can lead to a notable buildup of ammonia in the blood, a condition known as hyperammonemia (Kim et al., 2017; Dai et al., 2022). Additionally, the reactions orchestrated by GS and glutamate dehydrogenase (GDH) contribute significantly to the removal of ammonia, thus averting its toxic effects within other tissues (Spinelli et al., 2017). Any ammonia that escapes hepatic metabolism enters the systemic circulation, where it is detoxified by various tissues, including skeletal muscle, through the synthesis of glutamine (Gln) (Häussinger, 1986; Holecek, 2014). Hyperammonemia may ensue as a consequence of acute or chronic liver and kidney dysfunction (Cardoso et al., 2018; Jayalakshmi and Bernal, 2020). As hepatic UCEs and GS play pivotal roles in ammonia detoxification (Figure 2A), it is not surprising that hepatic deletion of GS triggers systemic hyperammonemia, symptoms commonly associated with cerebral damage, increased locomotion, impaired fear memory, and slightly reduced life span (Qvartskhava et al., 2015; Hakvoort et al., 2017).

### Hyperammonemia and relevant diseases

Healthy adults typically have ammonia concentrations in plasma ranging from 0 to 50 μM (Spinelli et al., 2017). Newborns exhibit levels between 50 μM and 150 μM (Spinelli et al., 2017), while patients experiencing hyperammonemia may have concentrations reaching up to 1.0 mM (Dasarathy et al., 2017). In adult patients, hepatic encephalopathy arises as a consequence of hyperammonemia resulting from liver failure. The symptom of hepatic encephalopathy is reversible when ammonia levels return to normal, provided that irreversible cerebral edema is absent (Häussinger et al., 2022). In children, a variety of inherited or acquired conditions, such as urea cycle disorders, can lead to hyperammonemia (Leonard and Morris, 2002; Stone et al., 2023). In liver dysfunction, ammonia accumulates and crosses the blood–brain barrier, leading to neurological impairment. Ammonia exerts harmful effects through multiple mechanisms, including glutamate (Glu) excitotoxicity, disruption of astrocyte function, oxidative stress, impaired energy metabolism, and neuroinflammation. These mechanisms collectively contribute to neuronal dysfunction, cerebral edema, and cognitive deficits observed in hepatic encephalopathy. Additionally, ammonia-induced alterations in neurotransmission and synaptic function further exacerbate the neurological manifestations of this condition. Understanding and targeting these mechanisms is crucial for developing effective therapeutic strategies to mitigate the harmful effects of ammonia in hepatic encephalopathy (Jayakumar and Norenberg, 2018). A recent study also reveals that elevated ammonium (NH_4_^+^) levels impair erythropoiesis by inducing oxidative stress, leading to defective erythroid maturation and impaired recovery from anemia (Lyu et al., 2024). Similarly, in β-thalassemia, excessive oxidative stress inactivates GS via protein oxidation, causing ammonia and Glu buildup, reduced Gln-to-Glu ratios, and ineffective erythropoiesis.

### Ammonia toxicity to the brain

In pathological conditions such as hepatic encephalopathy, high levels of ammonia play a central role in neurotoxicity. As the human brain lacks the capability to conduct the urea cycle, the primary pathway for ammonia removal within the central nervous system (CNS) is mediated by the astrocytic enzyme GS, which synthesizes Gln from ammonia and Glu. Consequently, heightened ammonia levels lead to an increased presence of Gln within CNS cells. This ammonia-induced increase in Gln has osmotic activity, contributing to cytotoxic edema through astrocyte swelling (Braissant et al., 2013). In cases of acute liver failure, glutamatergic neurons may be affected by Gln entrapment within neighboring astrocytes, resulting in increased neuroinhibition and diminished excitatory transmission (Desjardins et al., 2012). Furthermore, the astrocytic GS pathway, in conjunction with the secondary release of Glu into the intercellular space due to astrocyte swelling, can lead to the demise of glutamatergic neurons (Hertz and Kala, 2007). GDH reaction converting alpha-ketoglutarate (α-KG) to Glu can also reduce ammonia levels, and this process leads to the depletion of α-KG from the tricarboxylic acid cycle (Spinelli et al., 2017). The developing brain is particularly susceptible to elevated ammonia levels, which can lead to profound cognitive deficits, seizures, and the development of cerebral palsy (Enns, 2008). Neonates and infants experiencing hyperammonemia often exhibit cortical atrophy, ventricular enlargement, and demyelination, as well as hypodensities in gray and white matter regions (Gropman et al., 2007; Enns, 2008; Tuchman et al., 2008). When the development of the brain is immature or exposed to high ammonia levels for an extended duration, irreversible brain damage can occur (Bachmann, 2003; Enns, 2008; Tuchman et al., 2008). Elevated ammonia levels can impact all areas of the brain, potentially resulting in cerebral edema due to compromised osmoregulation. CNS edema can further lead to symptoms such as hyperventilation, respiratory alkalosis, apnea, and even fatality (Norenberg et al., 2005; Tuchman et al., 2008).

Ammonia toxicity in the brain is a complex phenomenon involving multiple molecular mechanisms. Liver diseases or metabolic disorders can lead to hyperammonemia, which has detrimental effects on brain function (Braissant et al., 2013; Dasarathy et al., 2017).

*Glu excitotoxicity*. Excess ammonia can lead to the accumulation of Glu in the brain, which in turn can overstimulate Glu receptors, leading to excitotoxicity. This can result in neuronal damage and cell death (Braissant et al., 2013).

*Disruption of astrocyte function*. Ammonia has been shown to disrupt the function of astrocytes, the most abundant glial cells in the brain. This disruption can impair the astrocytes’ ability to buffer extracellular potassium and neurotransmitters, leading to altered neuronal excitability and contributing to neurotoxicity (Dasarathy et al., 2017).

*Oxidative stress*. Ammonia can induce oxidative stress in the brain by promoting the production of reactive oxygen species (ROS) and impairing antioxidant defense mechanisms. This oxidative stress can damage cellular components and contribute to neuroinflammation and neuronal injury.

*Impaired energy metabolism*. High levels of ammonia can interfere with mitochondrial function and energy metabolism in brain cells, leading to decreased adenosine triphosphate (ATP) production and metabolic dysfunction, which can further exacerbate neuronal damage (Braissant et al., 2013).

*Neuroinflammation*. Ammonia can trigger neuroinflammatory responses in the brain, leading to the activation of microglia and the release of pro-inflammatory cytokines, which can contribute to neurotoxicity and neuronal dysfunction.

These mechanisms collectively contribute to the toxic effects of high ammonia levels on the brain, leading to cognitive impairment, neurological symptoms, and in severe cases, hepatic encephalopathy. Understanding these mechanisms is crucial for developing targeted therapeutic strategies to mitigate the detrimental effects of ammonia toxicity on brain function (Dasarathy et al., 2017).

## Novel roles of ammonia in the tumor microenvironment or cancer

In the context of cancer, ammonia has been found to accumulate in the tumor microenvironment (TME), where ammonia serves a dual role within the realm of metabolism; it functions as both a metabolic byproduct and a pivotal source of nitrogen that spans the biosphere (Kappler et al., 2017; Spinelli et al., 2017). This accumulation supports the biosynthesis of nonessential amino acids (NEAAs) and stimulates the activation of mechanistic target of rapamycin complex 1 (mTORC1), which in turn promotes tumor growth (Dai et al., 2022). Moreover, ammonia acts as a signaling molecule to stimulate lipid synthesis via sterol regulatory element-binding proteins (SREBPs), further supporting the metabolic demands of proliferating cancer cells (Cheng et al., 2022; Dai et al., 2022), as well as impairing various immune cell functions (Bell et al., 2023).

### Ammonia accumulates in the TME

A comparison of ammonia concentrations across various cancer types and their respective cell lines is presented in Table 1. Ammonia levels range from 1.5 mM to 5 mM, with hepatocellular and hepatoblastoma cancers showing higher concentrations (up to 5 mM) in models featuring c-Met/ΔN90-β-catenin and YAP/ΔN90-β-catenin, respectively (Dai et al., 2022). Breast cancer models show a broader range in ammonia concentrations (1–5 mM) across ER+ and BT-474 cell lines (Spinelli et al., 2017; Lie et al., 2019). Colon cancer and colorectal adenocarcinoma have similar ammonia ranges (2–4 mM) in HCT116 and HT-29 cell lines, respectively, while non-small cell lung cancer exhibits ammonia levels from 3 mM to 5 mM in H1975 cells (Lie et al., 2019). These variations suggest that the ammonia concentration may be differentially regulated or utilized depending on the cancer type and cellular context. Accumulation of ammonia within the liver TME is likely due to the inadequate vascularization of tumors, systemic inhibition of UCEs, including CPS1 and arginase 1 (ARG1), and high cell proliferation-elevated ammino acid catabolism, rendering it a distinctive niche for ammonia metabolism in human body (Figure 2B). As a consequence of ammonia accumulation in the liver TME, the production of Glu and NEAAs including alanine (Ala), aspartate (Asp), proline (Pro), and serine (Ser), as well as many urea cycle-related metabolites, increases (Figure 2B).

**Table 1:** A comparison of ammonia concentrations across various cancer types.

Cancer type	Ammonia concentration	Cancer model	Cell line	Reference
Hepatocellular carcinoma	1.5–5 mM	c-Met/ΔN90-β-catenin		Dai et al. (2022)
Hepatoblastoma	2.5–5 mM	YAP/ΔN90-β-catenin		Dai et al. (2022)
Breast cancer	1–4 mM	Subcutaneous xenografts	ER+	Spinelli et al. (2017)
Colon cancer	2–4 mM	Subcutaneous xenografts	HCT116	Eng et al. (2010)
Non-small cell lung cancer	3–5 mM	Subcutaneous xenografts	H1975	Eng et al. (2010)
Colon cancer	2.5–4 mM	Subcutaneous xenografts	Colo205	Eng et al. (2010)
Breast cancer	3.5–5 mM	Subcutaneous xenografts	BT-474	Eng et al. (2010)
Colorectal adenocarcinoma	2–4 mM	Subcutaneous xenografts	HT-29	Eng et al. (2010)

### Ammonia serves as substrates to promote NEAA production, stimulate mTORC1 activation, and promote tumor growth

**Figure 3:**
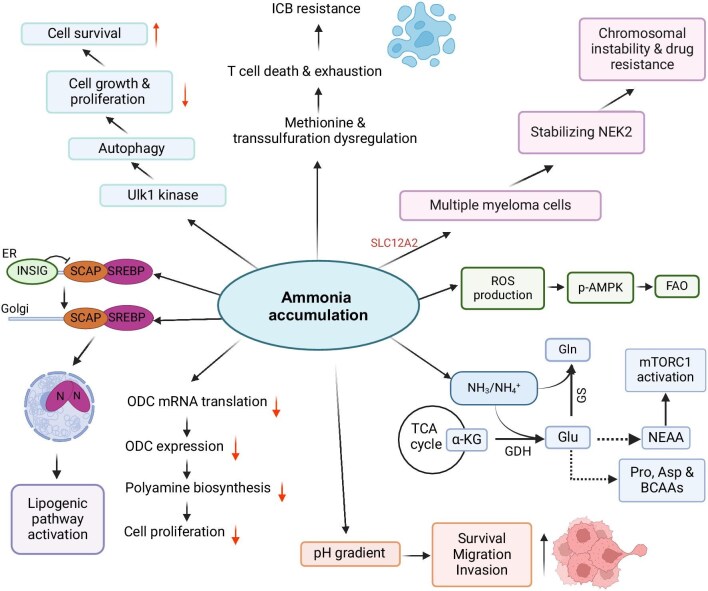
Effects of ammonia accumulation on cancer and immune cells. BCAA, branch chain amino acid; ICB, immune checkpoint blockade. Created in BioRender.

NEAAs play an important role in the synthesis of many nitrogenous compounds, including protein and nucleotide. Therefore, metabolic adaptation toward *de novo* synthesis of NEAAs may be demanded by proliferative cells when the cellular demand exceeds the local NEAA availability (Figure 3; Kurmi and Haigis, 2020). For example, increased transaminase activity and increased NEAA amounts were observed in MCF7 and T47D cells cultured with cell culture medium supplemented with ammonium chloride (Spinelli et al., 2017). Furthermore, studies have demonstrated that in human breast cancer, GDH serves as the primary mechanism for ammonia assimilation, because Glu serves as substrates for Pro, Asp, and Gln synthesis (Spinelli et al., 2017). Since NEAAs derived from Glu are linked to cellular proliferation and the development of tumors (Dai et al., 2022), the metabolic recycling of ammonia could facilitate the accelerated proliferation of breast cancer. ^15^N-labeled ammonium was used to perform stable isotope tracing, demonstrating that the amount of ^15^N-labeled Glu in the liver was significantly higher in the mice of GS ablation compared to wild-type mice (Dai et al., 2022). Furthermore, ^15^N-labeled NEAAs including Ala, tyrosine, and Asp, which could be derived from Glu, were also elevated by hepatic GS ablation (Dai et al., 2022). In mice, ammonia accumulates in the TME and is directly utilized to produce amino acids through GDH activity (Spinelli et al., 2017). An excessive level of ammonia can suppress the majority of cell proliferation and lead to cell apoptosis or necrosis because of its toxicity. Interestingly, it has been demonstrated that ammonia acts not only as a waste product but also as a crucial nitrogen source supporting tumor growth.

The coordination between nitrogen source availability and cellular growth is governed by the eukaryotic nitrogen-sensing pathway regulator, mTORC1, which oversees the regulation of enzymes involved in the metabolic recycling of ammonia, such as GDH, GS, and CPS1 (Lie et al., 2019; Kurmi and Haigis, 2020). The translocation of mTORC1 to the lysosome for activation is facilitated by the availability of key amino acids, such as arginine (Arg), Gln, leucine, lysine, and methionine (Met), signaling anabolic processes in cells (Kurmi and Haigis, 2020). NEAAs generated from Gln through glutaminolysis stimulate mTORC1 activation, and Glu-derived NEAAs, including Ala, Asp, Ser, Pro, glycine, and Gln, can also stimulate mTORC1 in various cancer cell lines (Figure 3; Spinelli et al., 2017; Meng et al., 2020; Krall et al., 2021; Dai et al., 2022). As previously noted, GDH-mediated Glu synthesis induces amino acid production and cellular growth (Li et al., 2019). Moreover, mTORC1 activation can be stimulated by ammonia alone and further enhanced by exogenous Gln supplementation with the concurrent ablation of GS (Dai et al., 2022).

### Ammonia serves as a signaling molecular to stimulate SREBP cleavage and promote lipid synthesis 

Lipids constitute the fundamental framework of both the plasma membrane and the membranes of all cellular organelles, making the acquisition of sufficient lipids an essential requirement for cell growth and proliferation (Cheng et al., 2018a, b). Levels of lipid are mainly regulated by the SREBP family, with SREBP-1 showing pronounced activation in malignancies (Eberlé et al., 2004). The activation of SREBPs follows a tightly controlled endoplasmic reticulum (ER)–Golgi–nucleus translocation process (Goldstein and Brown, 2015; Brown et al., 2018). The formation of an SREBP–SREBP cleavage-activating protein (SCAP) complex, resulting from the binding of SREBPs to SCAP, facilitates the transportation of this complex from the ER to the Golgi via COPII-coated vesicles (Sun et al., 2007; Brown et al., 2018). In the Golgi, Site-1 and Site-2 proteases sequentially cleave SREBPs, releasing N-terminal forms that translocate into the nucleus, where they activate the expression of lipogenic genes (Cheng et al., 1999; Espenshade et al., 1999). Nevertheless, the insulin-inducible gene protein (Insig or INSIG) impedes the trafficking of the SCAP–SREBP complex by binding to SCAP (Yabe et al., 2002; Yang et al., 2002).

It has been demonstrated that Gln and glucose are required for activation of SREBPs and lipogenesis. In contrast, the cleavage of SREBP-1 and SREBP-2 is induced by ammonia released from glutaminolysis in the presence of glucose (Figure 3; Cheng et al., 2022). The translocation of SREBP-1 into the nucleus is induced by ammonia in the presence of glucose but in the absence of Gln (Cheng et al., 2022). This underscores the capability of Gln-released ammonia to activate SREBPs and initiate lipogenesis. Moreover, SCAP–Insig dissociation can be dramatically activated by Gln or ammonia compared to glucose alone (Cheng et al., 2023). The trafficking of the complex to the Golgi is stimulated by both Gln and ammonia (Cheng et al., 2022, 2023). Additionally, the introduction of ammonia can restore the trafficking of the SREBP–SCAP complex, which was impaired by glutaminase inhibition (Cheng et al., 2022, 2023). This implies that the dissociation of *N*-glycosylated SCAP from Insig, stimulated by ammonia released from Gln, plays a crucial role. The binding of ammonia to SCAP induces sequential conformational changes, leading to the activation of SCAP–Insig dissociation, ultimately resulting in the translocation of SCAP–SREBP, SREBP activation, and lipogenesis, thereby promoting tumor growth (Cheng et al., 2022).

### Ammonia accumulation impairs various immune cell functions

**Figure 4:**
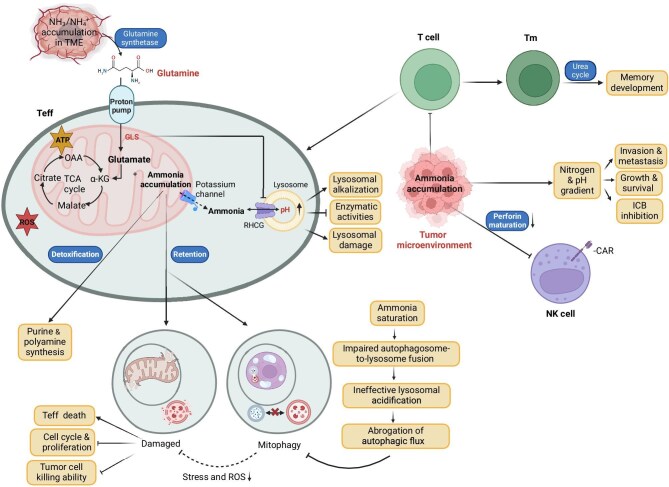
Pathways involved in the generation of ammonia in effector CD8^+^ T cells and the resultant cell damage and death. OAA, oxaloacetic acid; RHCG, Rh family C glycoprotein; mtDNA, mitochondrion DNA; Tm, memory T cell; Teff, effector T cell. Created in BioRender.

Ammonia possesses cytotoxic properties and has the potential to curtail cellular longevity. Elevated levels of ammonia in the TME can diminish T cell activation and proliferation, leading to T cell exhaustion (Figures 3 and 4; Bell et al., 2023). T cells exhibit rapid proliferation and possess a heightened metabolic demand for Met, a crucial component for driving T cell differentiation and proliferation (Bell et al., 2023). Met plays a pivotal role, as it can be transformed into *S*-adenosylmethionine, a common methyl donor. Tumors interfere with Met metabolism, reducing *S*-adenosylmethionine levels in CD8^+^ T cells, thereby compromising T cell immunity. The transsulfuration pathway is essential for converting Met to cysteine, the limiting substrate for glutathione synthesis (Bell et al., 2023). The decrease in transsulfuration flux caused by ammonia results in lowered glutathione levels, linked to increased ROS production (Figure 4; Bell et al., 2023).

Furthermore, both cancer cells and T cells exhibit a diminished capacity to clear ammonia compared to normal colon cells or hepatocytes, mainly due to the substantial downregulation of the urea cycle (Bell et al., 2023). The vulnerability of T cells, resulting from reduced transsulfuration flux combined with their limited ability to eliminate externally produced ammonia, creates a specific metabolic susceptibility (Bell et al., 2023). Within colorectal cancer, hepatocyte nuclear factor-4${\mathrm{\alpha }}$ activity and the downstream genes associated with ammonia clearance experience robust inhibition, culminating in the accumulation of extracellular ammonia (Figure 3; Bell et al., 2023). This heightened extracellular ammonia level detrimentally impacts the antitumor functionality of T cells by impeding their growth and inducing exhaustion.

To prevent ammonia toxicity, long-living CD8^+^ memory T cells mobilize the carbamoyl phosphate (Butterworth et al., 2018) metabolic pathway to clear ammonia, thus promoting memory development. CD8^+^ memory T cells use β-hydroxybutyrylation to upregulate CPS1 and trigger the carbamoyl phosphate metabolic cascade to form Arg in the cytosol. This cytosolic Arg is then translocated into the mitochondria where it is split by ARG2 to urea and ornithine. Cytosolic Arg is also converted to nitric oxide and citrulline by nitric oxide synthases. Thus, both the urea and citrulline cycles are employed by CD8^+^ memory T cells to clear ammonia and enable CD8^+^ T cell memory development (Tang et al., 2023). Different from CD8^+^ memory T cells with high expression of UCEs to help clear ammonia for their survival, rapidly proliferating effector T cells allow ammonia accumulation to mediate their death (Zhang et al., 2024). Mechanistically, glutaminolysis-derived ammonia is deposited in lysosomes and triggers the death of effector CD8^+^ T cells by promoting lysosomal alkalization, mitochondrial swelling, and further mitochondria disintegration (Figure 4; Zhang et al., 2024). It was also demonstrated that inhibiting glutaminolysis or blocking lysosomal alkalization prevents ammonia-induced effector T cell death and enhances T cell-based antitumor immunotherapy (Zhang et al., 2024).

Apart from T cells, natural killer (NK) cells also play a crucial role against malignant cells (Figure 4). It has been demonstrated that the antitumor activity of NK cells is suppressed by a cancer-conditioned medium (Domagala et al., 2025). Furthermore, the cytotoxicity of NK cells, as well as the efficacy of treatments involving antibody-based and chimeric antigen receptor (CAR)-NK cells, is impaired by ammonia accumulates in the cancer-conditioned medium and TME (Domagala et al., 2025). The decreased level of perforin leads to inhibition of NK cell activity, resulting from ammonia lysosomotropic features and its capacity to increase pH in acidic compartments (Lopez et al., 2012; Voskoboinik et al., 2015). Consequently, exposure to ammonia leads to a decrease in the mature form of perforin in NK cells, resulting in their impaired function (Domagala et al., 2025). These findings suggest that ammonia not only supports tumor growth by supplying nitrogen but also facilitates tumor evasion by acting as an immune checkpoint inhibitor for NK cells.

Recent research also elucidated a critical role of ammonia in the development of bortezomib resistance in multiple myeloma (MM) (Zhu et al., 2024). The study identified *Citrobacter freundii*, a gut microbiota species, as a contributor to increased circulating ammonium levels in MM patients. Elevated ammonium levels are linked to bortezomib resistance due to its entry into MM cells via the sodium/potassium/chloride transporter, solute carrier family 12 member 2 (SLC12A2). Once inside the cells, ammonium enhances the stability of the never in mitosis gene A-related kinase 2 (NEK2) protein, which is associated with chromosomal instability and contributes to drug resistance. This discovery highlights a novel mechanism by which the gut microbiota influences MM progression and treatment response, emphasizing the importance of the TME in therapeutic resistance (Zhu et al., 2024). Furthermore, therapeutic interventions targeting this ammonium-mediated resistance mechanism have shown promise. The loop diuretic furosemide was shown to downregulate SLC12A2, thereby reducing ammonium uptake by MM cells and improving bortezomib efficacy. Additionally, the probiotic *Clostridium butyricum* was found to alleviate bortezomib resistance, indicating that modulating the gut microbiota could be a viable strategy to enhance drug sensitivity. These findings suggest that targeting gut microbial nitrogen recycling and cellular ammonium uptake offers new avenues for overcoming drug resistance in MM. By integrating these approaches, it may be possible to develop more effective treatment strategies and improve patient outcomes in MM (Zhu et al., 2024).

### Ammonia accumulation suppresses polyamine biosynthesis

Recent study by Li et al. (2019) demonstrated that the accumulation of ammonia within cells significantly suppresses polyamine biosynthesis, an essential process for cell proliferation, which is particularly evident when examining the regulation of ornithine decarboxylase (ODC), the rate-limiting enzyme in polyamine biosynthesis. Ammonia directly restrains the activity of ODC, leading to decreased levels of putrescine, a polyamine necessary for cell growth (Li et al., 2019). It has been found that p53, a tumor suppressor gene, represses genes involved in the urea cycle, such as CPS1, ornithine transcarbamylase (OTC), and ARG1, resulting in increased ammonia levels within the cell and, consequently, a reduction in ODC activity (Li et al., 2019). Notably, the addition of exogenous ammonia or the depletion of UCEs causes a reduction in ODC protein levels but not in ODC mRNA levels, suggesting a post-transcriptional regulation of ODC by ammonia (Li et al., 2019). Moreover, this ammonia-induced decrease in ODC protein levels is not attributed to protein degradation through the ubiquitin–proteasome system, illustrated by the fact that ammonia still reduces ODC protein expression even in the presence of proteasome inhibitors (Li et al., 2019). Further investigation through polysome profiling has revealed that ammonia exposure leads to a reduction in ODC mRNA translation, highlighting a previously underappreciated capacity of ammonia to modulate protein synthesis at the translational level (Li et al., 2019). These findings offer a nuanced view of how ammonia regulates polyamine biosynthesis through the modulation of ODC activity, with potential implications for cell proliferation and tumor growth. When cellular mechanisms to eliminate ammonia are compromised, the resulting persistent high levels of ammonia can cause a halt in cell proliferation, pointing to a delicate balance in ammonia levels that cells must maintain for proper growth and function (Li et al., 2019).

### Ammonia derived from glutaminolysis plays a critical role in regulating autophagy

It is revealed that ammonia produced from glutaminolysis plays a critical role in regulating basal autophagy in both transformed and nontransformed human cells (Eng et al., 2010). This regulation is not due to nutrient depletion or inhibition of the mTOR but rather is a direct consequence of ammonia production during the conversion of Gln to α-KG in mitochondria (Eng et al., 2010). The addition of α-KG to cell cultures reduced ammonia production and consequently decreased the autophagy-inducing activity of conditioned cell medium (CCM), indicating that Gln is the primary source of autophagy-inducing ammonia (Eng et al., 2010).

Furthermore, it has been highlighted that ammonia, as a byproduct of glutaminolysis, not only contributes to anabolic metabolism but also accumulates under certain conditions to trigger autophagy. Indeed, the ammonia concentrations in interstitial fluids from human tumor xenografts are comparable to that required to induce autophagy *in vitro*, suggesting its role as a stress-protective agent through autophagic flux stimulation in actively proliferating cells (Eng et al., 2010; Li et al., 2016).

The underlying mechanism by which ammonia stimulates autophagic flux remains to be fully elucidated. It has been indicated that ammonia supports basal autophagy in a manner independent of mTORC1, known to be inhibited under nutrient deprivation or ATP depletion conditions. Instead, both CCM- and ammonia-induced autophagy were found to be sensitive to the depletion of Unc-51-like autophagy activating kinase 1 (Ulk1), suggesting that ammonia might act downstream of mTORC1 and upstream of Ulk1 in the autophagy pathway (Ganley et al., 2009; Eng et al., 2010; Thoreen et al., 2020). This proposes a model where Ulk1 and ammonia might play complementary roles in autophagosome formation and fusion with lysosomes, respectively, highlighting the need for further research to understand the interaction between Gln metabolism and autophagy regulation (Ganley et al., 2009; Eng et al., 2010).

### Ammonia stimulates ROS production and fatty acid oxidation

Apart from promoting NEAA production, an elevation in ammonia levels instigates the production of ROS in the context of CPS1 deficiency, which in turn activates the adenosine monophosphate-activated protein kinase (AMPK) pathway, as evidenced by the augmented levels of phosphorylated AMPK (p-AMPK) (Kim et al., 2017; Nitzahn and Lipshutz, 2020; Wu et al., 2021). The activation of AMPK is associated with the upregulation of CPT1C, which is crucial for the fatty acid oxidation (FAO) process within mitochondria (Wu et al., 2021). The enhancement of FAO is significant, as it supplies vital ATP for cellular proliferation and increases the resistance of hepatocellular carcinoma cells to chemotherapy, thereby promoting tumor growth and survival (Wu et al., 2021).

## Conclusion and future directions

In summary, under physiological conditions, mammals convert toxic ammonia into urea via the urea cycle to prevent neurotoxicity (DeBerardinis and Cheng, 2010; Tapper et al., 2015). Deficiencies in UCEs may result in hyperammonemia, leading to severe neurological deficits. In cancer, ammonia accumulates in the TME, promotes NEAA production, and stimulates mTORC1 activation, which in turn promotes tumor growth. Moreover, ammonia acts as a signaling molecule to stimulate lipid synthesis via SREBPs (Cheng et al., 2022; Dai et al., 2022). Ammonia's role in immunity has also been uncovered, showing it to be toxic to T cells, particularly memory T cells, thereby impairing the immune response against tumors (Tang et al., 2023). Additionally, ammonia influences polyamine biosynthesis by inhibiting ODC, affecting cell proliferation (Li et al., 2019). Furthermore, cancer cells manipulate intracellular pH through ammonia to create a favorable growth environment, contributing to disease progression and invasion (Silberman et al., 2019). Lastly, ammonia produced from glutaminolysis is implicated in regulating autophagy in human cells, independent of the mTOR pathway, suggesting a protective role in cellular stress responses (Eng et al., 2010). This multifaceted role of ammonia underscores its importance in physiological metabolism and disease pathology and warrants further investigation to understand its full biological impact.

Some future directions for exploration are raised as follows. (i) Although it is well-known that elevated levels of ammonia are harmful for brain and neural cells, the molecular mechanisms have not been completely uncovered (Gallego-Duran et al., 2024). (ii) It is not clear how and which NEAA(s) stimulate mTORC1 activation (Meng et al., 2020; Dai et al., 2022). (iii) Ammonia binds to SCAP to activate SREBPs in lipid metabolism, but the direct binding site has not been determined. Also, it is worth investigating whether ammonia similarly acts as a critical signaling molecule in other metabolisms as in lipid metabolism (Cheng et al., 2022). (iv) Extracellular ammonia accumulation induces T cell death, exhaustion, and immunosuppressive markers. Therefore, ammonia detoxification is a potential method to assist in immunotherapy (Zhang et al., 2024). (v) The mechanism by which ammonia suppresses ODC mRNA translation to inhibit the biosynthesis of polyamine and cell proliferation is unclear (Li et al., 2019). Meanwhile, ammonia can be used as an alternative nitrogen source to promote the proliferation of certain cancer cells. This discrepancy across cancer types worths further investigation.

## Notes

**Author contributions:** G.C., C.W., S.H., S.Y., Q.S., and Y.W. drafted the article. G.C. wrote the majority parts of the draft. W.D. conceptualized, wrote, and critically revised the article. All authors have read and approved the final version of the manuscript.
